# Comparing the Happiness Effects of Real and On-Line Friends

**DOI:** 10.1371/journal.pone.0072754

**Published:** 2013-09-03

**Authors:** John F. Helliwell, Haifang Huang

**Affiliations:** 1 Canadian Institute for Advanced Research and Vancouver School of Economics, University of British Columbia, Vancouver, British Columbia, Canada; 2 Department of Economics, University of Alberta, Edmonton, Alberta, Canada; Institut Pluridisciplinaire Hubert Curien, France

## Abstract

A recent large Canadian survey permits us to compare face-to-face (‘real-life’) and on-line social networks as sources of subjective well-being. The sample of 5,000 is drawn randomly from an on-line pool of respondents, a group well placed to have and value on-line friendships. We find three key results. First, the number of real-life friends is positively correlated with subjective well-being (SWB) even after controlling for income, demographic variables and personality differences. Doubling the number of friends in real life has an equivalent effect on well-being as a 50% increase in income. Second, the size of online networks is largely uncorrelated with subjective well-being. Third, we find that real-life friends are much more important for people who are single, divorced, separated or widowed than they are for people who are married or living with a partner. Findings from large international surveys (the European Social Surveys 2002–2008) are used to confirm the importance of real-life social networks to SWB; they also indicate a significantly smaller value of social networks to married or partnered couples.

## Introduction

There are constant changes in the types of activities that people engage in, and in the technologies they use to establish and enjoy their social connections. For example, Robert Putnam’s analysis of movements in social capital in the United States over the 20th century showed that memberships in most US organizations, the frequency of dinner parties, league bowling, and many other types of social connection grew for the first 70 years of the 20th century and declined thereafter [1]. Some commentators and researchers argued that there were new types of social connection, possibly more effective in nature, that were growing and possibly offsetting the effects of declines elsewhere. One of the key examples offered was the substitution of on-line for face-to-face (we use this term interchangeably with ‘real-life’) friendships. The internet could thereby be seen as providing ways of enhancing or replacing face-to-face friends through the availability of on-line social networks.

How can the effects of these differing trends be compared? To judge the importance and value of differing forms of friendship requires a common basis for valuation. The broadening availability of data for subjective well-being offers one possible solution to this valuation problem. If it were possible to measure each individual’s network of on-line and real-life friends, then their respective contributions to subjective well-being could provide a way of comparing their values, and hence to judge whether the quality of social networks as a whole was growing or shrinking. Only very recently has there been a survey that provided comparable measures of networks of face-to-face and on-line friends, set in the context of a well-being survey of sufficient size and scope to permit comparable assessments of the two types of friends.

## Literature Review

Friends and family are a long-established support for subjective well-being. Friends matter to happiness both for being potential sources of social support and for the pleasures from time spent together, whether at work, at play, or in activities for the benefit of others. Data from the Gallup World Poll suggest that having someone to call on in times of trouble is associated with a life evaluation that is higher, on a 0 to 10 scale, by almost half a point (page 298 in [2]). This is more than the equivalent of increasing household incomes by 150%. There is also a dose-response relationship, so that having more friends is better than having fewer. Evidence from the Canadian General Social Survey shows that, compared to respondents having no close friends, to have 3 to 5 close friends is associated with life satisfaction 0.24 points higher on a 10-point scale, an amount that rises to.32 for those with 6 to 10 close friends, and to 0.43 points for those with more than 20 close friends [3]. Also notable is that happiness depends not just on the number of close friends, but also the frequency with which they are seen [3], [4]. The same survey also asks about the number of close relatives, and the frequency with which they are seen. An interesting difference appears between friends and family. The number of close family matters more than the number of close friends, about twice as much up to 15 in number, with no gain thereafter, while frequency of seeing family contributes only half as much as the frequency of seeing close friends [3]. A similar result is found in US and other Canadian data analyzed by [5], where it is shown that the frequency of seeing friends adds twice as much to subjective well-being as does the frequency of seeing family. The US and Canadian surveys in [5] also reveal a strong relation between subjective well-being and the frequency of seeing friends, with those seeing friends most frequently having subjective well-being higher by 0.5 points on a ten-point scale.

All of these results are based on fully specified models with many other control variables, although there is no doubt likely to be some remaining element of mutual causality between subjective well-being and the frequency of seeing friends. For example, those who are at the bottom end of the subjective well-being scale, and especially those who are clinically depressed, often reduce the extent to which they reach out to friends. Indeed social withdrawal is a key element in the Beck Depression Inventory (BDI) [6], as supported in subsequent factor-analytic work by [7]. Thus some of the strong positive linkages between friends and happiness may reflect causal influences running in both directions. This is likely to apply for both real-life and on-line friends, and hence should not affect our comparisons in this paper between these two types of friends.

There are few studies of the linkages between on-line friendships and subjective well-being. One study [8] found a positive relation between subjective well-being and number of Facebook friends among a sample of 391 college-age subjects. Another study of college-age respondents in the United States, while not directly investigating the links between Facebook usage and subjective well-being, did find evidence that Facebook usage was correlated with proxy measures of social capital, but only for those with relatively low levels of satisfaction with campus life [9]. An earlier study of social capital and internet usage in a sample of US adolescents [10] found no significant relation between subjective well-being and time spent on-line. Those who spent more time messaging with close real-life friends were happier. Conversely, the relation between on-line time and subjective well-being was negative for those in contact with strangers or purely on-line friends. A recent study of Egyptian students found no significant relation between life satisfaction and intensity of Facebook usage [11].

Although there are many studies showing the effects of marital status on subjective well-being, we have not found previous attempts to see if the happiness effects of either real-life or on-line friends differ by marital status. Using two different surveys, we look for, and find, a large interaction effect in the happiness effects of marital status and real-life friends, but no significant differences for the effects of on-line friends.

We think that our results are the first to compare the happiness effects of real-life and on-line friends. Hence there are no directly comparable prior studies. Based on a meta-analysis [12] of fifty years of studies showing significantly more effective cooperation in conflict resolutions using face-to-face rather than written communications, we might conjecture that a similar difference might exist to differentiate the happiness effects of real-life and on-line friends.

## Data and Summary Statistics

The primary dataset for the paper is the 2011 Happiness Monitor survey sponsored by Coca-Cola and conducted in Canada between January 20 and 31, 2011 by Leger Marketing, using their online panel LegerWeb. The sample includes 5,025 Canadian residents, aged 16 and over, drawn from all ten Canadian provinces. The survey focuses on subjective well-being, and has questions that cover self-evaluation of life and other questions that can be used to construct alternative measures of well-being. It also has questions on people’s opinions about how various elements in life contribute to happiness. A section called *Canadiana* has occasionally light-hearted questions such as what is the happiest job in Canada, with a list that includes Zamboni driver and lumberjack.

From our perspective, the most interesting questions (other than the ones on well-being) are those on the size of social networks, separately for real-life friends and on-line friends. This presents an opportunity for us to examine potential differences between these two types of networks, specifically in their contributions to subjective well-being.

We use regression analysis to relate measures of subjective well-being to the sizes of social networks, as well as income and demographic controls. We will also use control variables to pick up differences in personalities; such variables include self-reported stress, time spent exercising and contributions to charitable causes.

**Figure 1:**
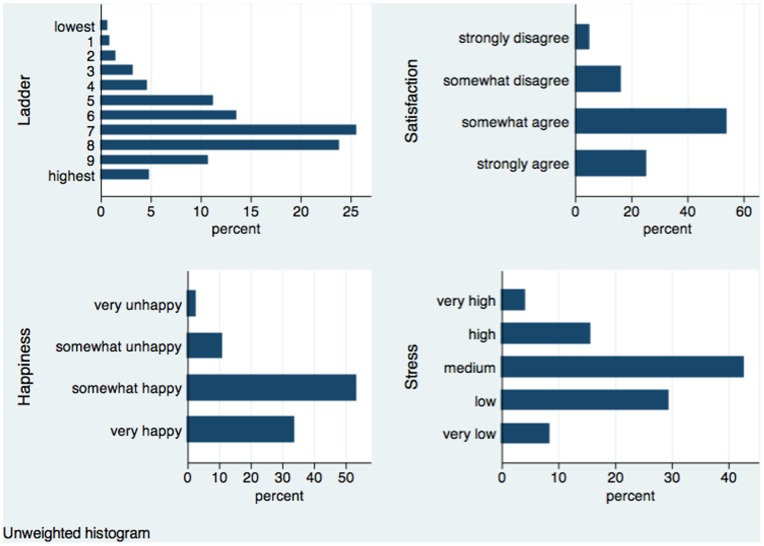
Distribution: Subjective well-being and stress in the Happiness Monitor survey.

The survey’s primary measure of subjective well-being is an 11-point (from 0 to 10) *life ladder*, based on the question “Please imagine a ladder with steps numbered from zero at the bottom to 10 at the top. The top of the ladder represents the best possible life for you and the bottom of the ladder represents the worst possible life for you. On which step of the ladder would you say you personally feel you stand at this time?” This question, also known as Cantril’s Self-Anchoring Ladder, is frequently used in well-being studies, including the recent World Happiness Report [13] and many studies cited therein. We plot the distribution of sample responses in the first panel of Figure 1. The mode is “7” with a quarter of the respondents. The next greatest concentration is “8” with about 20% of the responses. The sample mean is 6.8, significantly lower than for the Canadian ladder responses in the Gallup World Poll, as shown in figure 2.3 of the World Happiness Report.

**Figure 2:**
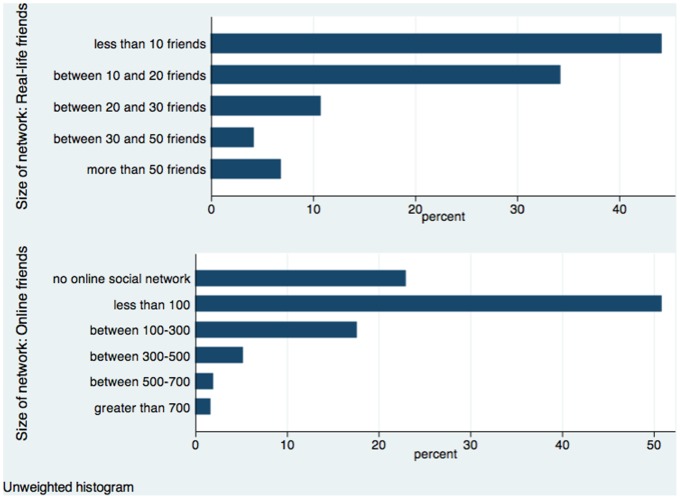
Distribution: Size of social networks in the Happiness Monitor survey.

It is possible to construct two other measures of well-being from the survey. One is life satisfaction, based on the four-point responses to the question “To what extent do you agree with each of the following statements” that include a statement “I am satisfied with my life”. The four points are “strongly agree”, “somewhat agree”, “somewhat disagree” and “strongly disagree”. The second panel of Figure 1 shows the distribution. The mode, covering more than 50% of the responses, is “somewhat agree”. Another potential measure is the response to the question “How happy are you at the beginning of 2011? Very happy, somewhat happy, somewhat unhappy, very unhappy.” The distribution of happiness is similar to that of life satisfaction: the third step “somewhat happy” has more than 50% of the sample. We will use these two measures of well-being for robustness tests.

There is also a question on the level of stress, specifically the response to the question “How would you rate your average daily stress levels? Very low, Low, Medium, High, Very high.” Its distribution is shown in the last panel of Figure 1. The response of “Medium” has the greatest share of responses at 40%.

We now move on to the two questions on social networks. The first question concerns real-life friends. The exact wording is “How big is your real-life social network of friends?” The permitted responses, unless the respondents refuse to answer, include “Less than 10 friends”, “Between 10 and 20 friends”, “Between 20 and 30 friends”, “Between 30 and 50 friends”, and “More than 50 friends”. The distribution of the network size is shown in the upper panel of Figure 2. A large majority of the sample, almost 80%, is in the first two categories (i.e., with fewer than 20 friends).

The immediately next question in the survey concerns online friends: “How big is your online social network?” The responses include “I don't have an online social network”, “Less than 100”, “Between 100–300”, “Between 300–500”, “Between 500–700” and -Greater than 700”. The distribution is shown on the lower panel of Figure 2. A large majority of the sample either has no online friends (about 25%) or have fewer than 100 of them (about 50%).

The two network questions have different numbers of steps, and both have some steps with sparse responses (see Figure 2). We correct for these problems by combining the top two categories of real-life network into one single category with 11% of the sample, and the top three categories of online network into one category with 9% of the sample. This way, we turn the two network sizes into a comparable scale of four steps. In the case of real-life network, the four categories are “less than 10”, “10–20”, “20–30” and “30 or more”, with 44%, 34% 11% and 11% of the sample, respectively. The size of online network falls into “0”, “1–100”, “100–300”, “300 or more”, with 23%, 50.8%, 17.6% and 8.6% of the sample, respectively.

Table 1 presents summary statistics of other variables. The average age is 45. Forty-five percent (45%) of the sample are married; 15% in common-law relation, 5% dating, 23% single; the remaining 12% are divorced, separated, widowed or are unknown. The income information is based on categorical responses of income intervals. We estimate the midpoint of each interval under the assumption that income follows a log-normal distribution. We then assign respondents in each interval the corresponding midpoint estimate. The categories for the income variables are “$20,000 and below”, “$20,001 to $35,000”, “$35,001 to $50,000”, “$50,001 to $75,000”, “$75,001 and $110,000” and “more than $110,000”. The estimated midpoints are $13,605, $27,073, $41,895, $60,345, $87,895 and $136,849 respectively. About 15% of the sample did not provide income information. We use a dummy variable to indicate such a status in the regression analysis. Among those that have valid income information, the sample mean is $51 thousand. The average time spent on moderate to high intensity exercising is 1.78 hours per week. Close to 60% of survey respondents indicated that they currently volunteer or give time or money to charitable causes.

**Table 1:** Summary statistics - weighted sample in the Happiness Monitor survey.

Variable	Mean	Std. Dev.	Min.	Max.	N
Age	44.93	16.77	16	85	5025
Male	0.49	0.5	0	1	5025
Marital status: married	0.45	0.5	0	1	5025
Marital status:common-law	0.15	0.36	0	1	5025
Marital status: dating	0.05	0.22	0	1	5025
Marital status: single	0.23	0.42	0	1	5025
Marital status: divorced/separated/widowed	0.12	0.32	0	1	5025
Income; thousands	51.24	33.92	13.6	136.85	4270
Unemployed	0.05	0.22	0	1	5025
High school or below	0.21	0.41	0	1	4979
Some post-secondary	0.24	0.43	0	1	4979
University degrees	0.55	0.5	0	1	4979
Exercise per week; hours	1.78	1.73	0	5	4975
Average stress levels;0 to 1	0.46	0.23	0	1	4998
Volunteer or contributeto charity	0.59	0.49	0	1	5025

**Figure 3:**
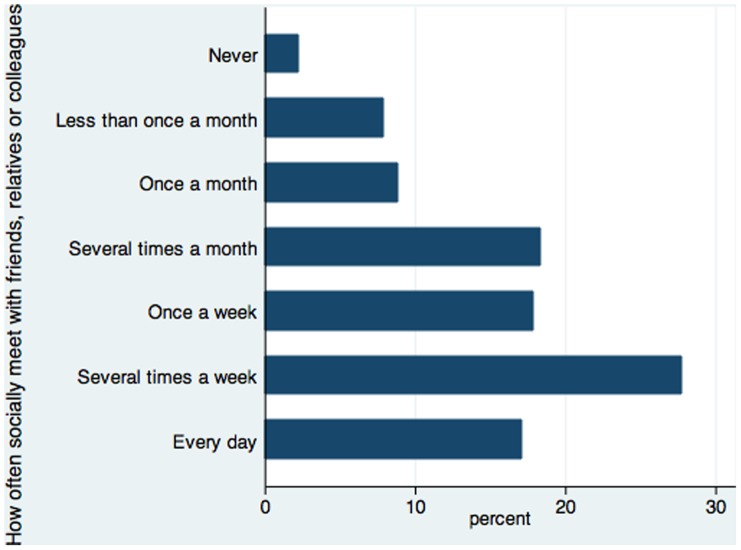
Distribution: Socially meeting with friends, etc. in the ESS.

A second dataset that we use is the European Social Survey (ESS), a biennial cross-sectional survey of residents aged 15 and over within private households that is “designed to chart and explain the interaction between Europe's changing institutions and the attitudes, beliefs and behaviour patterns of its diverse populations” (The European Social Survey project). We use the cumulative file for rounds 1–4 (2002, 2004, 2006, 2008) that has 34 participating countries. The ESS does not have information relating to online social networks. Instead, it has information on survey respondents’ frequency of *socially* meeting with friends, relatives or colleagues. Figure 3 plots the distribution of the frequency, in the categories of “Never”, “Less than once a month”, “Once a month”, “Several times a month”, “Once a week”, “Several times a week” and “Every day”.

**Figure 4:**
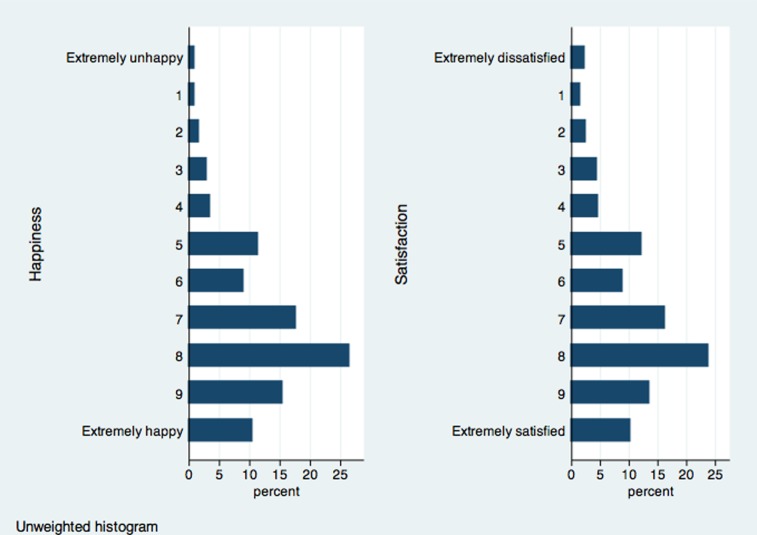
Distribution: Subjective well-being in the ESS.

The ESS has two alternative measures of SWB, happiness and life satisfaction. The two underlying questions are “Taking all things together, how happy would you say you are?” and “All things considered, how satisfied are you with your life as a whole nowadays?”. Both SWB measures are on a 11-point ascending scale from 0 to 10, with 0 indicating extremely unhappy/dissatisfied and 10 indicating extremely happy/satisfied. Figure 4 plots the distributions. Table 2 presents summary statistics of other variables.

**Table 2:** Summary statistics - weighted sample in the ESS.

Variable	Mean	Std. Dev.	Min.	Max.	N
Age	45.65	18.27	15	123	183860
Male	0.47	0.5	0	1	184745
Marital status: married	0.56	0.5	0	1	184988
Marital status: civil partnership(waves 3 and 4)	0.01	0.12	0	1	94142
Marital status: single	0.27	0.44	0	1	184988
Completed high school	0.34	0.48	0	1	184988
Some post-secondary	0.03	0.16	0	1	184988
Completed university	0.27	0.44	0	1	184988
Labor force status: unemployed	0.04	0.21	0	1	184988
Labor force status: doing paid work	0.51	0.5	0	1	184988
Trust: most people can be trusted (scale 0–10)	4.54	2.5	0	10	184154
How often attendingreligious services (scale 0–6)	1.61	1.58	0	6	183862
Self-reported generalhealth (scale 1–5)	3.65	0.92	1	5	184718
Income Decile 1	0.08	0.27	0	1	126395
Income Decile 2	0.09	0.28	0	1	126395
Income Decile 3	0.11	0.31	0	1	126395
Income Decile 4	0.13	0.34	0	1	126395
Income Decile 5	0.12	0.33	0	1	126395
Income Decile 6	0.11	0.31	0	1	126395
Income Decile 7	0.1	0.3	0	1	126395
Income Decile 8	0.09	0.28	0	1	126395
Income Decile 9	0.11	0.32	0	1	126395
Income Decile 10	0.06	0.24	0	1	126395

By covering many different countries, adopting a different way of measuring interactions with friends, and by having additional measures of subjective well-being, the ESS increases the power and generality of our findings about the happiness effects of real-life friends.

## Regression Analysis

Our regression analysis estimates the following equation
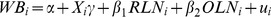
(1)where the dependent variable 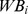 is the measure of well-being for respondent 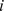. The right-hand side includes an intercept 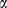, a vector of control variables in 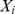, as well as 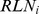, the size of real-life network of friends, and 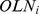, the size of online network. The control vector includes age, gender and marital status, education, income and unemployment status. To help remove possible effects of unmeasured personality differences, we also include the time spent on exercise, whether the respondent volunteers or contributes to charitable causes and self-reported daily stress levels. These variables are likely to be influenced by individual personality differences, as are the size of networks of friends. If our key results for the effects of friends hold whether or not our equations include these other variables, they we are more confident in concluding that our results are not being driven by personality differences.

The measure of life ladder is ordinal; but as commonly found in the literature, little is lost if we treat it as cardinal. For example, [14] reported that the choice between probit regressions, which treats dependent variables as ordinal, and Ordinary Least Squares (OLS), which treats dependent variables as cardinal, makes virtually no difference to the estimated relationships between happiness and important explanatory variables. In this paper, we will present results from the method of OLS; Ordered Probit estimations yield qualitatively similar findings. In terms of quantitative evaluations, our discussions will be based largely on the concept of compensating differentials: we will compare the estimated effects of social networks to the estimated effects of income. The ratios of coefficients are almost invariant to the choice of regression methods, as switching between OLS and Ordered Probit affects estimated coefficients almost proportionally (see [15] for an example).

The variables of interest on the right-hand side are the sizes of social networks in real life and on-line. In both cases, the size information is based on categorical choices from a set of intervals (the distributions are shown in Figure 2). We enter the size information into the regressions in two different ways. The first approach uses a set of dummy variables to indicate the intervals. This avoids making assumptions regarding the functional form of the relationships between network sizes and subjective well-being. The second approach imposes an assumption that the relationship is log-linear. To implement the log-linear approach, we turn the intervals into continuous values by assigning the midpoint of an interval to observations in that interval. In the case of real-life friends, the category “Less than 10 friends” is assigned a value of 5, the category “Between 10 and 20 friends” receives a value of 15, and so on. The top category “More than 50 friends” is assigned a value of 60. Similarly, we assign continuous values to the size of online network by assigning zero to the category “I don’t have an online social network”, the value of 50 to “Less than 100”, the value of 200 to “Between 100–300”, and so on. The top category “Greater than 700” receives a value of 800.

Table 3 shows the regression output. In all columns, the dependent variable is the 0–10 point life ladder. In the first column, we enter the network sizes as a set of categorical variables. In the second column, the network sizes are in (logged) continuous values. The first two columns show the happiness effects of real-life and on-line friends without the inclusion of other variables. In columns (3) and (4) we add a full set of control variables to be described below, and in columns (5) and (6) we further test the robustness of our findings by adding a measure of psychological stress.

**Table 3:** Regressions with *life ladder* as the dependent variable - the Happiness Monitor survey.

	(1)	(2)	(3)	(4)	(5)	(6)
Real-life friends: 10–20	0.5		0.29		0.26	
	(0.07)***		(0.07)***		(0.06)***	
Real-life friends: 20–30	0.71		0.32		0.29	
	(0.1)***		(0.1)***		(0.09)***	
Real-life friends: 30 or more	0.67		0.36		0.3	
	(0.12)***		(0.11)***		(0.11)***	
Online friends: 1–100	−.31		−.15		−.10	
	(0.07)***		(0.07)**		(0.06)	
Online friends: 100–300	−.69		−.18		−.17	
	(0.09)***		(0.1)*		(0.09)*	
Online friends: 300 or more	−.81		−.21		−.15	
	(0.13)***		(0.14)		(0.13)	
Size of network: Real-life friends		0.33		0.19		0.16
		(0.04)***		(0.04)***		(0.04)***
Size of network: Online friends		−.13		−.04		−.03
		(0.02)***		(0.02)**		(0.01)**
Logged income			0.43	0.43	0.45	0.45
			(0.06)***	(0.06)***	(0.06)***	(0.06)***
Income unknown			0.14	0.13	0.12	0.11
			(0.08)	(0.08)	(0.08)	(0.08)
Male			−.16	−.17	−.21	−.22
			(0.06)***	(0.06)***	(0.06)***	(0.06)***
Age			−.08	−.08	−.06	−.06
			(0.01)***	(0.01)***	(0.01)***	(0.01)***
Age squared/100			0.1	0.1	0.08	0.08
			(0.01)***	(0.01)***	(0.01)***	(0.01)***
Marital status: married			0.75	0.74	0.76	0.76
			(0.1)***	(0.1)***	(0.09)***	(0.09)***
Marital status: common-law			0.7	0.7	0.67	0.68
			(0.12)***	(0.12)***	(0.11)***	(0.11)***
Marital status: dating			0.65	0.64	0.68	0.67
			(0.19)***	(0.19)***	(0.18)***	(0.18)***
Marital status: single			0.29	0.28	0.3	0.29
			(0.13)***	(0.12)**	(0.12)**	(0.12)**
Some post-secondary			−.002	−.001	0.02	0.02
			(0.09)	(0.09)	(0.09)	(0.09)
University degrees			0.05	0.05	0.07	0.07
			(0.08)	(0.08)	(0.07)	(0.07)
Unemployed			−1.07	−1.07	−1.03	−1.03
			(0.18)***	(0.18)***	(0.17)***	(0.17)***
Exercise per week; hours			0.08	0.08	0.06	0.06
			(0.02)***	(0.02)***	(0.02)***	(0.02)***
Volunteer or contribute to charity			0.36	0.36	0.35	0.35
			(0.06)***	(0.06)***	(0.06)***	(0.06)***
Average stress levels; 0 to 1					−2.24	−2.24
					(0.13)***	(0.13)***
Obs.	4489	4489	4428	4428	4416	4416
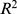	0.03	0.03	0.17	0.17	0.24	0.24

Notes: Standard errors in parentheses. *, **, and *** indicate statistical significance at 90 percent, 95 percent and 99 percent levels.

Columns (1) and (2) provide the simplest and starkest evidence that real-life and on-line friends have very different associations with subjective well-being. Whether measured as categories or as continuous variables, real-life friends are positively associated with happiness, while on-line networks have a negative relationship. The strong positive effects of real-life networks are consistent with much other research. The strong negative effects of on-line friends are more surprising. The difference between the two effects is striking. Because the size and nature of friendships is likely to be correlated with age, gender, marital status, income and other variables, we shall do our main analysis of results using columns (3) and (4), which confirm our first results showing sharply differing effects of real-life and on-line friends, but largely eliminate the estimated negative effects of on-line networks.

The estimated effects of the newly added control variables are largely consistent with the literature. As commonly found, there is a positive and statistically significant income effect. The estimates of the effect are largely invariant across specifications, and suggest that doubling income (*i.e.*, an increase of logged income by 0.7 unit) increases the life ladder by about 0.3. Later we will use this estimate as a benchmark to evaluate the quantitative importance of social networks. In term of genders, male respondents tend to report a lower evaluation of life. There is a U-shape relationship between age and life ladder: the life ladder falls as age rises but makes a U-turn in the 40 s. In terms of marital status, the happiest respondents are those who are in a relationship (married, common-law, or dating). The least happy group, which we use as the reference group, includes those who are divorced, widowed or separated. The group of non-dating singles lies in between. The difference between singles and the in-relationship groups is substantial, equivalent to the impact of increasing logged income by an entire unit. There is a strong negative effect associated with being unemployed, a positive effect associated with exercising and volunteering time or money for charitable causes. The estimated coefficients on the educational variables turn out largely insignificant, likely because the control variables already include measure of household income and social-context variables that are correlated with education.

The sizes of social networks enter columns (3) and (5) in Table 3 as categorical variables of intervals. The reference groups that are left out are those that have the smallest networks, specifically “less than 10” in the case of real-life friends and zero in the case of online friends. The estimated effects of real-life friends are all statistically significant and quantitatively substantial. Compared to the group that has fewer than 10 friends, the estimates in column (3) suggests that having 10–20 friends increases the life ladder by 0.29 points, equivalent to the improvement associated with a 0.7 unit of logged income (or 100% increase of income). Compared to the same reference group, having 20–30 friends increases the ladder by 0.32 points, while having more than 30 friends increases the evaluation by 0.36. The estimates thus suggest a substantial non-linearity in the relationship between network sizes and well-being. The most substantial increase in well-being occurs when moving from the group of “less than 10” to the group of “10 to 20”. The marginal contribution beyond that is quite small. Columns (4) and (6), which treat network sizes as continuous values, also show positive and significant coefficients. The variable of network size is expressed in logarithms. The coefficient estimate is 0.19, equivalent to the well-being gain from a 0.44 rise increase in logged income. Doubling the number of real-life friends is equivalent to increasing income by more than one half.

The findings for on-line networks are strikingly different from those for real-life friends. Compared to the reference group that has no on-line network at all, having a greater number of on-line friends is not associated with a higher level of life ladder. If anything, the correlation is negative, generally not significant at the 95% confidence level. Column (4) uses logged continuous values to express the size of networks. In such a specification, the estimated effect from the online network is negative and significant, although it is quantitatively small (doubling the number of online friends has the equivalent effect of reducing income by 10%).

The regressions described above estimate the effect of the online social network while controlling for the size of real-life network. Given the positive correlations between online networks and real-life networks (the correlation coefficient is 0.25), we expect the coefficients for online networks size to become more positive if we remove the variables for real-life networks from the regressions. We performed this test using the equation of column (3), and it did indeed make the coefficients on online network size less negative, but they still maintain a negative sign throughout (though none of them has statistical significance at the 95% level).

The final two columns of the table add to the right-hand side of the regressions an extra variable: the self-reported level of daily stress. The inclusion of the stress variable increases the r-squared substantially (from 17% to 24%), but has little impact on the estimated effects of network sizes; nor does it change the contrast between the two types of network. These findings reinforce our earlier point that omitted variables, including those correlated with personality, will not put our conclusions at risk as long as their inclusion does not alter the key coefficients, and especially the relative impact of on-line and real-life friends. The equations adding stress thus provide additional evidence of the robustness of our results.

The next table, or Table 4, uses the four-step life satisfaction and happiness answers as alternative dependent variables. For better comparability with the 0–10 point life ladder, we rescale the two variables so that they, too, have zero for the lowest level of satisfaction/happiness and 10 for the highest level. The estimates are similar to those from the estimations based on the life ladder. Real-life networks are important to satisfaction and happiness, while online networks are largely irrelevant. The biggest difference is that the estimated effect of real-life friendship is even greater for happiness and life satisfaction than for the life ladder. In the case of life ladder in Table 3, doubling the number of real-life friends has the same effect as increasing income by more than one-half (exp.44 = 1.55). For life satisfaction, doubling the number of friends is equivalent to a doubling of income (exp.69 = 1.99), while for happiness it has the same effect as a trebling of income (exp 1.12 = 3.06).

**Table 4:** Regressions with alternative measures of SWB: *Life satisfaction* and *happiness* - the Happiness Monitor survey.

	Satisfaction	Satisfaction	Happiness	Happiness
	(1)	(2)	(3)	(4)
Real-life friends: 10–20	0.51		0.54	
	(0.09)***		(0.09)***	
Real-life friends: 20–30	0.67		0.69	
	(0.14)***		(0.12)***	
Real-life friends: 30 or more	0.48		0.7	
	(0.15)***		(0.14)***	
Online friends: 1–100	−.16		−.13	
	(0.1)		(0.1)	
Online friends: 100–300	−.18		0.02	
	(0.14)		(0.13)	
Online friends: 300 or more	−.30		0.09	
	(0.19)		(0.18)	
Size of network: Real-life friends		0.29		0.37
		(0.06)***		(0.05)***
Size of network: Online friends		−.04		−.004
		(0.02)*		(0.02)
Logged income	0.42	0.42	0.33	0.33
	(0.08)***	(0.08)***	(0.07)***	(0.07)***
Income unknown	0.08	0.08	−.04	−.05
	(0.11)	(0.11)	(0.11)	(0.11)
Male	−.07	−.08	−.20	−.21
	(0.09)	(0.09)	(0.08)***	(0.08)***
Age	−.11	−.11	−.08	−.09
	(0.02)***	(0.02)***	(0.02)***	(0.01)***
Age^2/100^	0.12	0.12	0.1	0.11
	(0.02)***	(0.02)***	(0.02)***	(0.02)***
Marital status: married	0.76	0.75	0.6	0.59
	(0.13)***	(0.13)***	(0.12)***	(0.12)***
Marital status: common-law	0.59	0.58	0.77	0.76
	(0.17)***	(0.17)***	(0.15)***	(0.15)***
Marital status: dating	0.08	0.06	0.56	0.56
	(0.25)	(0.25)	(0.23)**	(0.23)**
Marital status: single	−.25	−.27	−.01	−.02
	(0.17)	(0.17)	(0.16)	(0.16)
Some post-secondary	−.01	−.01	−.17	−.16
	(0.13)	(0.13)	(0.11)	(0.11)
University degrees	−.03	−.02	−.23	−.22
	(0.11)	(0.11)	(0.1)**	(0.1)**
Unemployed	−1.19	−1.19	−.78	−.78
	(0.24)***	(0.24)***	(0.23)***	(0.23)***
Exercise per week; hours	0.11	0.11	0.1	0.1
	(0.02)***	(0.02)***	(0.02)***	(0.02)***
Volunteer or contribute to charity	0.58	0.58	0.47	0.47
	(0.09)***	(0.09)***	(0.08)***	(0.09)***
Obs.	4663	4663	4722	4722
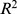	0.12	0.12	0.1	0.09

Notes: Standard errors in parentheses. *, **, and *** indicate statistical significance at 90 percent, 95 percent and 99 percent levels.

Next, we split the sample into two subgroups: one includes respondents who are married or in a common-law relationship; the other includes the rest of the sample. This is to compare the importance of friendship and social networks in the two segments of the population. Our earlier results have already shown that both marriage and real-life friends contribute importantly to subjective well-being, and by somewhat comparable amounts. Our results also show that those who are single but dating are almost as happy as those who are living together, once again suggesting the importance of the social aspects of co-habitation.

Partners in successful marriages often describe their spouses as their best friends. This suggests the possibility that human social needs and desires can be met to some extent either by spouses or by other friends, which in turn might suggest that those who are married might have less need for large networks of other friends. Perhaps they may also have less time available to build and maintain networks of other friends. Some combination of lower need and scarcer time is suggested by the new Canadian data for the number of friends. When the size of networks of friends is put on a scale ranging from zero to 1.0, its average value for the married and other cohabiting respondents is.36, significantly below the mean of.47 for all other respondents (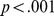). European Social Survey (ESS) data suggest that married respondents spend only two-thirds as much time with friends as do those who are not married (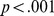). The Canadian General Social Survey, which also asks about the frequency of seeing friends, shows that time spent with friends is 30% less for married than unmarried respondents (from GSS 17, 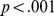).

Table 5 presents the split-sample estimates. Its first two columns use the life ladder as the dependent variable, while the other four apply to life satisfaction and happiness, respectively. For each of the alternative dependent variables, one column shows estimates from the married/partnered sample; the other shows estimates from the rest of the sample. The findings regarding social networks are similar across the measures of SWB. The sizes of on-line networks are largely statistically insignificant for both subgroups. The real-life networks, in contrast, have positive and generally significant effects on SWB; but there is a stark contrast between the married/partnered respondents and the rest of the sample. Real-life networks have greater effects for people who are not married/partnered. The estimated differences are substantial. In the case of life ladder, the estimated contribution of having more than more than 30 friends is 0.72 in the un-married/partnered sample; the standard error is 0.18. In contrast, the estimated contribution is only 0.14 for people who are married or in a common-law partnership; the standard error is 0.14. There is thus no overlap in the 95% confidence intervals of the two estimates. Regressions using the alternative measures of SWB show a similar pattern of difference, with real-life networks being significantly more valuable for people who are not married or in a common-law partnership. Finally, we note that when we separate the married group and the common-law group instead of treating them as a single sample, we find by and large similar relationships between real-life friends and happiness. Table 6 reports the estimates. In all cases, the point estimates of the real-life friends’ effects in the married sample or in the common-law sample are smaller than those in the rest of the population (in Table 5). This explains why we combine the married and the common-law population together as a single group, and compare it to the rest of the population.

**Table 5:** Split-sample estimations: respondents who are married/in common-law partnerships (sample A) v.s. the rest (sample B) - the Happiness Monitor survey.

	Ladder		Satisfaction		Happiness	
	A	B	A	B	A	B
Real-life friends: 10–20	0.12	0.55	0.38	0.74	0.4	0.77
	(0.08)	(0.12)***	(0.11)***	(0.17)***	(0.1)***	(0.15)***
Real-life friends: 20–30	0.04	0.8	0.36	1.23	0.42	1.16
	(0.11)	(0.17)***	(0.17)**	(0.24)***	(0.14)***	(0.22)***
Real-life friends: 30 or more	0.14	0.72	0.24	0.87	0.44	1.12
	(0.14)	(0.18)***	(0.18)	(0.26)***	(0.18)**	(0.22)***
Online friends: 1–100	−.20	−.004	−.19	−.05	−.15	−.03
	(0.08)**	(0.14)	(0.12)	(0.2)	(0.11)	(0.18)
Online friends: 100–300	−.16	−.17	−.10	−.24	−.05	0.14
	(0.11)	(0.18)	(0.17)	(0.26)	(0.16)	(0.24)
Online friends: 300 or more	−.20	−.18	−.49	−.14	0.05	0.18
	(0.21)	(0.21)	(0.26)*	(0.29)	(0.22)	(0.28)
Logged income	0.49	0.4	0.39	0.44	0.43	0.17
	(0.07)***	(0.1)***	(0.09)***	(0.13)***	(0.09)***	(0.12)
Income unknown	0.33	−.16	0.12	0.06	0.03	0.0006
	(0.09)***	(0.16)	(0.14)	(0.21)	(0.14)	(0.19)
Male	−.17	−.11	0.06	−.20	−.18	−.22
	(0.07)**	(0.11)	(0.1)	(0.15)	(0.1)*	(0.13)
Age	−.05	−.12	−.09	−.13	−.10	−.08
	(0.02)***	(0.02)***	(0.02)***	(0.03)***	(0.02)***	(0.02)***
Age squared/100	0.07	0.15	0.1	0.15	0.11	0.1
	(0.02)***	(0.02)***	(0.02)***	(0.03)***	(0.02)***	(0.02)***
Marital status: common-law	−.01		−.21		0.11	
	(0.09)		(0.13)		(0.12)	
Marital status: dating		0.68		0.31		0.72
		(0.2)***		(0.28)		(0.26)***
Marital status: single		0.37		0.005		0.17
		(0.14)***		(0.19)		(0.18)
Some post-secondary	−.02	0.03	−.06	0.05	−.10	−.24
	(0.11)	(0.16)	(0.15)	(0.22)	(0.13)	(0.19)
University degrees	0.06	0.11	−.006	0.007	−.26	−.15
	(0.09)	(0.15)	(0.12)	(0.2)	(0.12)**	(0.18)
Unemployed	−.62	−1.38	−.71	−1.59	−.39	−1.10
	(0.26)**	(0.25)***	(0.3)**	(0.35)***	(0.28)	(0.33)***
Exercise per week; hours	0.09	0.07	0.1	0.1	0.1	0.09
	(0.02)***	(0.03)**	(0.03)***	(0.04)**	(0.03)***	(0.04)**
Volunteer or contribute to charity	0.31	0.4	0.64	0.43	0.43	1
	(0.08)***	(0.11)***	(0.11)***	(0.16)***	(0.11)***	(0.14)***
Obs.	2858	1570	3008	1655	3037	1685
*R* ^2^	0.13	0.18	0.07	0.13	0.06	0.12

Notes: Standard errors in parentheses. *, **, and *** indicate statistical significance at 90 percent, 95 percent and 99 percent levels.

**Table 6:** Treating married respondents (sample M) and common-law respondents (sample C) as separate groups - the Happiness Monitor survey.

	Ladder		Satisfaction		Happiness	
	M	C	M	C	M	C
Real-life friends: 10–20	0.05	0.27	0.34	0.43	0.37	0.44
	(0.08)	(0.17)	(0.12)***	(0.24)*	(0.12)***	(0.2)**
Real-life friends: 20–30	0.05	−.07	0.35	0.34	0.29	0.96
	(0.13)	(0.25)	(0.19)*	(0.36)	(0.16)*	(0.32)***
Real-life friends: 30 or more	0.24	−.19	0.27	0.03	0.61	−.09
	(0.16)	(0.32)	(0.2)	(0.41)	(0.2)***	(0.37)
Online friends: 1–100	−.17	−.30	−.18	−.16	−.11	−.20
	(0.08)**	(0.2)	(0.13)	(0.29)	(0.12)	(0.26)
Online friends: 100–300	−.11	−.30	−.10	−.08	−.08	0.07
	(0.13)	(0.25)	(0.19)	(0.37)	(0.19)	(0.31)
Online friends: 300 or more	−.24	−.22	−.34	−.64	−.04	0.18
	(0.27)	(0.34)	(0.3)	(0.52)	(0.28)	(0.39)
Logged income	0.42	0.73	0.42	0.35	0.38	0.59
	(0.08)***	(0.15)***	(0.1)***	(0.2)*	(0.1)***	(0.17)***
Income unknown	0.3	0.31	0.06	0.33	−.05	0.38
	(0.1)***	(0.27)	(0.14)	(0.41)	(0.16)	(0.33)
Male	−.16	−.17	−.12	0.53	−.23	−.004
	(0.08)*	(0.17)	(0.12)	(0.23)**	(0.11)**	(0.18)
Age	−.04	−.08	−.09	−.11	−.09	−.12
	(0.02)	(0.04)**	(0.03)***	(0.06)**	(0.03)***	(0.05)***
Age squared/100	0.06	0.11	0.1	0.12	0.1	0.14
	(0.02)***	(0.04)***	(0.03)***	(0.06)**	(0.03)***	(0.05)***
Some post-secondary	−.10	0.18	−.19	0.24	−.27	0.28
	(0.11)	(0.24)	(0.16)	(0.31)	(0.15)*	(0.27)
University degrees	−.003	0.24	0.02	−.05	−.32	−.08
	(0.1)	(0.19)	(0.14)	(0.26)	(0.13)**	(0.23)
Unemployed	−.78	−.27	−.58	−.94	−.51	−.19
	(0.26)***	(0.51)	(0.35)*	(0.56)*	(0.35)	(0.48)
Exercise per week; hours	0.1	0.05	0.12	0.09	0.12	0.04
	(0.02)***	(0.05)	(0.03)***	(0.06)	(0.03)***	(0.06)
Volunteer or contribute to charity	0.42	0.09	0.69	0.56	0.59	0.05
	(0.09)***	(0.15)	(0.13)***	(0.22)**	(0.13)***	(0.19)
Obs.	2239	619	2373	635	2396	641
*R* ^2^	0.12	0.13	0.07	0.08	0.07	0.08

Notes: Standard errors in parentheses. *, **, and *** indicate statistical significance at 90 percent, 95 percent and 99 percent levels.

We also split the sample by gender (male and female) and by age group (16–34, 35–49, 50–64 and 65 and up). Table 7 presents the estimates. The estimated effects of on-line networks are mostly insignificant, or have signs indicating negative contributions to SWB. Real-life friends, on the other hand, have positive and mostly significant estimates. The biggest exception concerns the age group 35 to 49, for which none of the network variables (online or real) have any positive and significant effects. In fact, the highest size of on-line network is negatively associated with life ladder, with strong statistical significance.

**Table 7:** Regressions by population sub-groups with life ladder as the dependent variable - the Happiness Monitor survey.

	male	female	age1634	age3549	age5064	age65up
	(1)	(2)	(3)	(4)	(5)	(6)
Real-life friends: 10–20	0.28	0.29	0.39	0.21	0.19	0.32
	(0.09)***	(0.1)***	(0.15)***	(0.17)	(0.09)**	(0.13)**
Real-life friends: 20–30	0.31	0.33	0.37	0.13	0.38	0.29
	(0.13)**	(0.14)**	(0.19)**	(0.27)	(0.14)***	(0.17)*
Real-life friends: 30 or more	0.45	0.26	0.38	0.12	0.34	0.86
	(0.15)***	(0.17)	(0.22)*	(0.24)	(0.17)**	(0.22)***
Online friends: 1–100	−.25	−.04	−.09	−.21	−.18	−.02
	(0.09)***	(0.11)	(0.24)	(0.19)	(0.09)**	(0.12)
Online friends: 100–300	−.27	−.06	−.13	−.10	−.03	−.44
	(0.13)**	(0.15)	(0.23)	(0.2)	(0.15)	(0.26)*
Online friends: 300 or more	−.25	−.18	−.02	−1.00	0.003	0.31
	(0.19)	(0.21)	(0.26)	(0.33)***	(0.28)	(0.71)
Logged income	0.55	0.33	0.19	0.55	0.6	0.58
	(0.08)***	(0.09)***	(0.14)	(0.15)***	(0.08)***	(0.12)***
Income unknown	0.16	0.15	−.05	−.05	0.32	0.43
	(0.13)	(0.11)	(0.2)	(0.25)	(0.11)***	(0.14)***
Male			0.02	−.36	−.24	−.03
			(0.14)	(0.15)**	(0.08)***	(0.12)
Age	−.10	−.06	−.66	−.11	−.56	0.48
	(0.02)***	(0.02)***	(0.14)***	(0.35)	(0.3)*	(0.37)
Age squared/100	0.12	0.09	1.20	0.14	0.54	−.31
	(0.02)***	(0.02)***	(0.28)***	(0.42)	(0.26)**	(0.25)
Marital status: married	0.74	0.76	1.19	0.95	0.86	0.25
	(0.14)***	(0.14)***	(0.45)***	(0.31)***	(0.13)***	(0.15)
Marital status: common-law	0.73	0.7	1.29	0.84	0.8	0.37
	(0.17)***	(0.17)***	(0.46)***	(0.33)**	(0.18)***	(0.24)
Marital status: dating	0.82	0.53	0.95	0.65	1.04	0.38
	(0.29)***	(0.25)**	(0.48)**	(0.62)	(0.38)***	(1.62)
Marital status: single	0.27	0.32	0.67	0.62	0.24	0.19
	(0.18)	(0.18)*	(0.45)	(0.35)*	(0.17)	(0.27)
Some post-secondary	−.16	0.14	0.15	−.0002	−.06	−.02
	(0.12)	(0.13)	(0.2)	(0.24)	(0.12)	(0.16)
University degrees	0.02	0.07	0.5	−.05	−.02	0.09
	(0.11)	(0.11)	(0.21)**	(0.21)	(0.1)	(0.14)
Unemployed	−1.11	−1.00	−.89	−1.14	−1.18	2.11
	(0.25)***	(0.27)***	(0.35)**	(0.35)***	(0.23)***	(0.21)***
Exercise per week; hours	0.09	0.08	0.1	0.04	0.07	0.1
	(0.02)***	(0.03)***	(0.04)***	(0.04)	(0.02)***	(0.03)***
Volunteer or contribute to charity	0.34	0.37	0.46	0.32	0.31	0.23
	(0.09)***	(0.1)***	(0.13)***	(0.14)**	(0.1)***	(0.15)
Obs.	2391	2037	946	728	1854	900
*R* ^2^	0.21	0.14	0.12	0.16	0.19	0.12

Notes: Standard errors in parentheses. *, **, and *** indicate statistical significance at 90 percent, 95 percent and 99 percent levels.

Finally, we split the sample along the interactive gender×age groups: young (16–34) males and females, middle-aged (34–50) males and females, elder (50 and up) males and females. Table 8 presents the estimates. Many of the estimated effects of the real-life network become insignificant, likely due to the drop in sample size. But they retain their positive sign with very few exceptions. It is worth noting that, among middle-aged females, having the largest size of online network (300 online friends or more) has a large, negative and significant association with SWB. The estimated effect is so large that it exceeds that of being unemployed by a substantial margin. One possible explanation for this association is reverse causality, with unhappy people extending greater efforts to expand their on-line networks or resorting to more intensive online activity that leads to greater network sizes.

**Table 8:** Regressions by more population sub-groups with life ladder as the dependent variable - the Happiness Monitor survey.

	age1634M	age1634F	age3549M	age3549F	age50upM	age50upF
	(1)	(2)	(3)	(4)	(5)	(6)
Real-life friends: 10–20	0.31	0.5	0.26	0.13	0.27	0.2
	(0.23)	(0.19)***	(0.18)	(0.29)	(0.09)***	(0.11)*
Real-life friends: 20–30	0.3	0.47	0.11	0.4	0.46	0.26
	(0.28)	(0.27)*	(0.38)	(0.37)	(0.12)***	(0.17)
Real-life friends: 30 or more	0.42	0.36	0.27	−.12	0.57	0.48
	(0.31)	(0.3)	(0.3)	(0.37)	(0.17)***	(0.2)**
Online friends: 1–100	−.70	0.72	−.16	−.30	−.17	−.08
	(0.28)**	(0.4)*	(0.22)	(0.31)	(0.09)*	(0.12)
Online friends: 100–300	−.70	0.62	0.05	−.23	−.45	0.04
	(0.28)**	(0.38)*	(0.23)	(0.34)	(0.18)**	(0.18)
Online friends: 300 or more	−.55	0.65	−.71	−1.66	0.11	−.09
	(0.33)*	(0.4)*	(0.34)**	(0.68)**	(0.27)	(0.44)
Logged income	0.25	0.06	0.92	0.18	0.68	0.49
	(0.2)	(0.19)	(0.17)***	(0.23)	(0.09)***	(0.1)***
Income unknown	−.05	0.07	0.27	−.22	0.43	0.34
	(0.32)	(0.25)	(0.31)	(0.35)	(0.13)***	(0.12)***
Male						
Age	−.74	−.56	−.01	−.48	0.18	−.03
	(0.2)***	(0.22)**	(0.42)	(0.61)	(0.09)**	(0.09)
Age squared/100	1.34	1.01	0.02	0.58	−.11	0.06
	(0.4)***	(0.44)**	(0.5)	(0.73)	(0.07)	(0.07)
Marital status: married	1.11	1.81	0.62	1.17	0.72	0.56
	(0.58)*	(0.79)**	(0.37)*	(0.45)***	(0.14)***	(0.13)***
Marital status: common-law	1.12	1.97	1.01	0.61	0.59	0.65
	(0.6)*	(0.79)**	(0.4)**	(0.47)	(0.19)***	(0.21)***
Marital status: dating	1.12	1.37	0.96	0.34	1.30	0.57
	(0.65)*	(0.79)*	(1.07)	(0.87)	(0.35)***	(0.51)
Marital status: single	0.62	1.29	0.54	0.63	−.0006	0.28
	(0.59)	(0.79)	(0.39)	(0.58)	(0.21)	(0.19)
Some post-secondary	−.04	0.34	−.17	0.05	−.19	0.05
	(0.31)	(0.26)	(0.3)	(0.4)	(0.13)	(0.14)
University degrees	0.43	0.52	−.07	−.07	−.07	0.07
	(0.32)	(0.26)**	(0.26)	(0.35)	(0.11)	(0.12)
Unemployed	−.84	−.93	−.86	−1.37	−1.46	−.82
	(0.54)	(0.44)**	(0.39)**	(0.67)**	(0.27)***	(0.37)**
Exercise per week; hours	0.09	0.13	0.06	0.02	0.1	0.05
	(0.06)	(0.05)**	(0.05)	(0.07)	(0.02)***	(0.03)*
Volunteer or contribute to charity	0.67	0.28	0.2	0.38	0.21	0.39
	(0.2)***	(0.16)*	(0.17)	(0.24)	(0.1)**	(0.13)***
Obs.	409	537	465	263	1517	1237
*R* ^2^	0.15	0.12	0.19	0.17	0.26	0.13

Notes: Standard errors in parentheses. *, **, and *** indicate statistical significance at 90 percent, 95 percent and 99 percent levels.

## Findings from the ESS

The previous section makes three empirical observations: 1) the size of real-life social networks contributes positively to SWB; 2) the size of on-line social networks does not contribute to SWB; 3) the real-life social network is more valuable for respondents who are not married or in a common-law relationship. We can test the robustness of the first and the third observations using the European Social Survey (ESS), a large international survey whose first four rounds (2002–2008) include more than 180,000 individual respondents in 34 countries. The ESS does not, unfortunately, have information about on-line social networks.

We will use the ESS data to estimate equations similar to equ(1), but without the variable for on-line networks. There are two alternative measures of SWB from the survey, happiness and life satisfaction, both on the same 11-point scale from 0 to 10 as is used for the Cantril ladder in the Canadian survey. The variable of interest on the right-hand side is the response to the question “how often do you meet socially with friends, relatives or work colleagues?” This measure of social interactions is originally recorded in seven categories: “Never”, “Less than once a month”, “Once a month”, “Several times a month”, “Once a week”, “Several times a week” and “Every day”. To construct categorical indicators with sufficient sample sizes, we collapse the survey responses into five categories: “less than once a month including never” (with a combined mass of 11%), “once a month” (9%), “several times a month including once a week” (36%), “several times a week” (27%) and “every day” (17%). We then include the categorical indicators on the right-hand side of our estimations to explain SWB.

Our regressions also include a conventional set of control variables in SWB analysis: age, age squared, educational attainment, marital status, labour force status and income. We also control for country fixed effects and wave fixed effects (The wave 1 ESS was conducted in 2002, wave 2 in 2004, wave 3 in 2006 and wave 4 in 2008). The country fixed effects remove cross-country differences in per capita income as well as the potentially different interpretations regarding the scale of satisfaction and happiness. We also use the general level of trust (the response to the question whether “most people can be trusted, or that you can't be too careful in dealing with people”), the frequency of attending religious services outside special occasions, and self-reported health status to control for the differences in social and religious attitudes, subjective health, as well as possible personality differences. We use Ordinary Least Squares, clustering errors at the country level.

Tables 9 and 10 present the results. The findings for the control variables are similar to those reported in the previous section. Males tend to report lower happiness and satisfaction. There is a U-shape relation between age and SWB; those in the 40 s report the lowest happiness and life satisfaction. Compared to the divorced, separated or the widowed, being married or in a civil partnership is associated with higher SWB. The same is true for being never married, but to a lesser extent. Higher income is associated with higher SWB. We find positive income-SWB relation throughout the income distribution. The relation flattens out at middle and higher income, but the marginal contribution of income to well-being never falls to zero or becomes negative. In terms of labour force status, there is no significant difference between being employed and not participating. Being unemployed, however, is a significant negative factor with a large estimated effect. The SWB difference between unemployment and non-participation is similar to the difference arising from moving an individual from the lowest income decile to the 7th decile in the case of happiness, or to the 8th decile in the case of life satisfaction. General trust, the frequency of attending religious services and self-reported health status are all positive contributing factors to happiness and to life satisfaction.

**Table 9:** Regressions with happiness as the dependent variable - the ESS. Sample A includes only married/civiled partnered respondents; sample B includes the rest.

	Full sample	Sample A	Sample B
Friends etc - once a month	0.4	0.33	0.53
	(0.05)***	(0.06)***	(0.06)***
Friends etc - several times a month	0.65	0.62	0.72
	(0.04)***	(0.05)***	(0.09)***
Friends etc - several times a week	0.81	0.76	0.92
	(0.05)***	(0.05)***	(0.07)***
Friends etc - every day	0.98	0.89	1.11
	(0.05)***	(0.07)***	(0.07)***
Male	−.15	−.10	−.21
	(0.03)***	(0.02)***	(0.05)***
Age	−.05	−.04	−.06
	(0.004)***	(0.005)***	(0.005)***
Age^2/100^	0.05	0.05	0.06
	(0.003)***	(0.004)***	(0.004)***
Married	0.77	0.16	
	(0.04)***	(0.12)	
Civil partnership	0.57		
	(0.09)***		
Single	0.17		0.12
	(0.04)***		(0.04)***
Completed Highschool	0.005	−.03	0.03
	(0.04)	(0.06)	(0.03)
Some post-secondary	0.12	0.04	0.2
	(0.03)***	(0.05)	(0.04)***
Completed university	0.07	0.05	0.07
	(0.07)	(0.09)	(0.06)
Unemployed	−.65	−.77	−.53
	(0.09)***	(0.12)***	(0.08)***
Paid work	−.03	−.09	0.06
	(0.02)	(0.03)***	(0.03)**
Most people can be trusted	0.1	0.09	0.12
	(0.01)***	(0.02)***	(0.004)***
How often attend religious services	0.04	0.02	0.06
	(0.01)***	(0.01)**	(0.01)***
Self-reported health status	0.55	0.5	0.6
	(0.03)***	(0.03)***	(0.03)***
Income decile 2	0.3	0.24	0.33
	(0.08)***	(0.11)**	(0.11)***
Income decile 3	0.48	0.45	0.47
	(0.11)***	(0.09)***	(0.16)***
Income decile 4	0.55	0.56	0.5
	(0.12)***	(0.12)***	(0.13)***
Income decile 5	0.6	0.52	0.62
	(0.1)***	(0.11)***	(0.11)***
Income decile 6	0.67	0.63	0.66
	(0.08)***	(0.09)***	(0.1)***
Income decile 7	0.7	0.62	0.73
	(0.09)***	(0.1)***	(0.1)***
Income decile 8	0.77	0.69	0.82
	(0.05)***	(0.06)***	(0.11)***
Income decile 9	0.82	0.73	0.91
	(0.09)***	(0.1)***	(0.12)***
Income decile 10	0.85	0.75	0.95
	(0.09)***	(0.1)***	(0.12)***
Obs.	124069	67379	56690
*R* ^2^	0.24	0.21	0.26

Notes: Standard errors in parentheses.

* , **, and *** indicate statistical significance at 90 percent, 95 percent and 99 percent levels.

**Table 10:** Regressions with life satisfaction as the dependent variable - the ESS.

	Full sample	Sample A	Sample B
Friends etc - once a month	0.31	0.2	0.51
	(0.06)***	(0.08)**	(0.07)***
Friends etc - several times a month	0.57	0.5	0.7
	(0.06)***	(0.07)***	(0.06)***
Friends etc - several times a week	0.74	0.66	0.89
	(0.05)***	(0.06)***	(0.06)***
Friends etc - every day	0.83	0.64	1.07
	(0.06)***	(0.11)***	(0.05)***
Male	−.15	−.12	−.17
	(0.03)***	(0.03)***	(0.05)***
Age	−.07	−.07	−.07
	(0.005)***	(0.008)***	(0.007)***
Age^2/100^	0.08	0.07	0.08
	(0.005)***	(0.008)***	(0.006)***
Married	0.52	0.16	
	(0.04)***	(0.16)	
Civil partnership	0.38		
	(0.13)***		
Single	0.12		0.17
	(0.03)***		(0.03)***
Completed Highschool	−.05	−.10	0.01
	(0.06)	(0.08)	(0.05)
Some post-secondary	0.11	0.06	0.18
	(0.04)**	(0.04)	(0.05)***
Completed university	0.07	0.07	0.05
	(0.06)	(0.07)	(0.06)
Unemployed	−1.04	−1.07	−.99
	(0.12)***	(0.16)***	(0.11)***
Paid work	−.04	−.08	−.008
	(0.03)	(0.05)	(0.03)
Most people can be trusted	0.14	0.14	0.15
	(0.006)***	(0.009)***	(0.007)***
How often attend religious services	0.07	0.06	0.1
	(0.02)***	(0.02)***	(0.02)***
Self-reported health status	0.6	0.56	0.66
	(0.03)***	(0.03)***	(0.04)***
Income decile 2	0.29	0.31	0.27
	(0.08)***	(0.07)***	(0.12)**
Income decile 3	0.57	0.63	0.5
	(0.13)***	(0.1)***	(0.19)***
Income decile 4	0.66	0.72	0.63
	(0.14)***	(0.14)***	(0.15)***
Income decile 5	0.74	0.75	0.74
	(0.11)***	(0.13)***	(0.12)***
Income decile 6	0.89	0.86	0.94
	(0.11)***	(0.11)***	(0.14)***
Income decile 7	0.93	0.93	0.93
	(0.1)***	(0.11)***	(0.13)***
Income decile 8	1.03	1.03	1.05
	(0.1)***	(0.08)***	(0.15)***
Income decile 9	1.16	1.16	1.16
	(0.1)***	(0.11)***	(0.14)***
Income decile 10	1.25	1.24	1.25
	(0.13)***	(0.13)***	(0.19)***
Obs.	124087	67405	56682
*R* ^2^	0.26	0.25	0.27

Sample A includes only married/civiled partnered respondents; sample B includes the rest.

Notes: Standard errors in parentheses.

* , **, and *** indicate statistical significance at 90 percent, 95 percent and 99 percent levels.

Our variable of special interest on the right-hand side is the frequency of socially meeting with friends, relatives and colleagues. The estimated coefficients on this variable are all positive. A higher frequency is associated with greater happiness and satisfaction. For happiness, the greatest improvement occurs when moving away from the bottom (less than once a month) to the category of “once a month”; the happiness increment is 0.4 point. There is a further gain of 0.25 when moving to “several times a month”, then a further 0.16 gain to “several time a week”, then a further 0.17 gain to “every day”. For life satisfaction, the marginal improvements associated with the same step-by-step moves are 0.31, 0.26, 0.17 and 0.09, respectively in the same order. These contributions, especially those arising from a move from the bottom (less than once a month) to the next level (once a month), are very substantial, more than the SWB gain due to a jump from the 5th income decile to the top decile in the case of happiness, and equivalent to a jump from the 5th decile to the 8th decile in the case of life satisfaction. But it is important to realize that there is only about 10% of the population whose frequency of social interactions is at the bottom with less than once a month; so we are talking about moving away from a small minority that has a very low frequency of social interactions. If we focus on the move from “several times a month” to “several times a week”, the marginal contribution is more moderate. The income equivalent is a move from the 5th decile to the 8th in the case of happiness, and from the 5th to the 7th in the case of life satisfaction.

We now examine the difference between married couples/civil partners and those who are not in such relations. The findings from the Canadian survey indicate that the importance of real-life networks to SWB is greater for those who are not in a marriage or a common-law partnership. The ESS yields qualitatively similar observations. The second and the third columns of Tables 9 present estimates from the spilt-sample estimation, with happiness as the dependent variable. Table 10 has the same split-sample estimations with life satisfaction as the dependent variable. For both SWB measures, the estimated effects of social interactions are lower for married/partnered couples than for the rest of the population. In most cases, the differences between point estimates are greater than two standard errors of individual estimates.

The findings from the ESS thus confirm that real-life social networks (captured as the frequency of social interactions in the ESS) are positive and substantial contributing factors to SWB, with an importance that is greater for people who are not married or in a civil partnership.

## Conclusion

We have used data from a large new Canadian survey to estimate the subjective well-being benefits of comparably measured networks of real-life and on-line friends. We have three main results. First, we confirm many earlier studies showing the importance of real-life friends to subjective well-being. Second, we find that comparably measured networks of on-line friends have zero or negative correlations with subjective well-being, whether or not allowance is made for the influence of other factors. Third, we find significant interactions between marriage and friends as sources of happiness. The estimated well-being impact of the number of friends is much smaller for those who are married or living together, suggesting that friends and spouses provide some similar happiness benefits. We also find that single people who are dating have subjective well-being significantly higher than those who are not. The effect is almost as high as for living together, which in turn is nearly as high as being married. These results also suggest that the company and friendship of marriage matter as much as the legal institution. Our Canadian results on the well-being value of networks of real-life friends are confirmed also for large samples of data from the European Social Survey. We also confirm from the ESS the greater value of friends for those who are not married.

Our results on the relative values of real-life and on-line friends are likely to be specific to generations, countries, and demographic groups, and to change as social and technological changes alter the possibilities for these two types of social connection to be either mutually supportive or inconsistent in their consequences for well-being. The overall importance of friendship to the maintenance of subjective well-being would seem to support more widespread collection of comparable data on the size and quality of friendships of different types, whether real-life or on-line, or on or off the job.

The limitations of our current results relate in part to the fact that we have only one survey comparably measuring the size of networks of real-life and on-line friends, so that our results might depend to some extent on sample or population specifics. As in all correlation analysis, there are risks that the influences we treat as running from friends to happiness may also be running in the reverse direction, or be determined by some third factors not controlled for. Our hope is that these difficulties are sufficiently shared by the data for the two types of friends that our comparative results might be expected to hold in more experimental contexts. We hope at least to have provided a useful first look.
